# P-580. Hot Springs, Hot Zones: An Ecological Study on the Relationship Between Thermal Spring Density and Legionella Mortality

**DOI:** 10.1093/ofid/ofaf695.794

**Published:** 2026-01-11

**Authors:** Lilian Fung, Kevin Ikuta

**Affiliations:** University of California, Los Angeles, Los Angeles, CA; West Los Angeles VA, Los Angeles, California

## Abstract

**Background:**

Legionnaire’s disease, an atypical pneumonia caused by an aerobic gram negative bacillus Legionella pneumophila, remains a public health concern due to continued outbreaks. Global estimates suggest that Japan has among the highest legionella mortality rates in the world and some have hypothesized that this is due to the density of, and culture around, thermal hot springs and spas. In order to explore this hypothesis we performed an ecological study evaluating the relationship between the number of thermal springs and spas per capita and the morality rate of legionella by region.

Spas and Thermal Spring Density vs. Legionella Death Rate by Region

**Figure:**
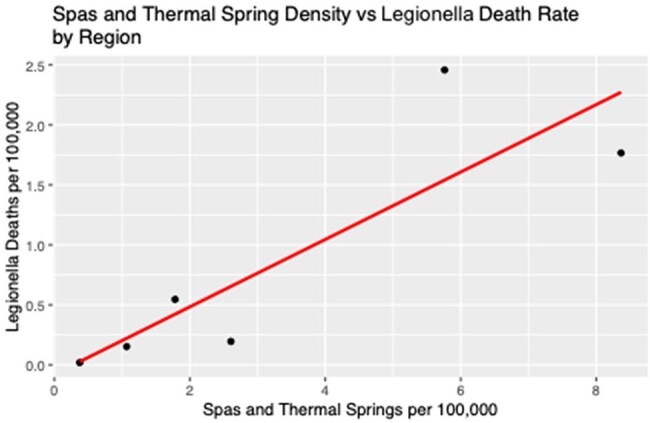

Legionella Death Rate for Top 20 Spa Revenue Countries vs. Others

**Figure:**
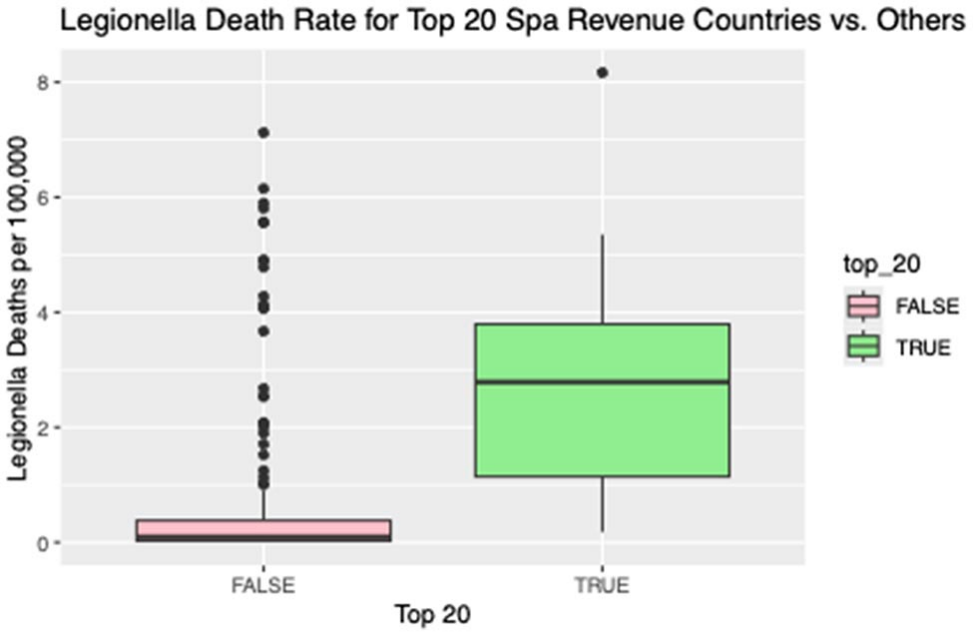

**Methods:**

The Global Wellness Institute is an industry research initiative that monitors the wellness industry. In 2018 they produced an estimate of the number of spas and thermal springs across six regions. Additionally, they produced an estimate of the top 20 countries in revenue from thermal springs in 2018. We extracted region and country level estimates of legionella mortality in 2019 as well as population estimates from the global burden of diseases study. We used a univariate gaussian linear regression model to evaluate the relationship between spas and thermal springs per 100 000 people and legionella mortality rate at the region level. Additionally, we performed a students T-test comparing the legionella mortality rates for countries in the top 20 versus those outside the top 20 in thermal springs revenue.

**Results:**

We found a statistically significant relationship between spa and thermal spring density and legionella mortality with our model estimating that for every increase in spa and thermal spring of 1 per 100,000 there was a commensurate increase in legionella mortality of 0.3 per 100,000 (95% CI of 0.12-0.44 and p < 0.05). [Figure 1] with a R2 of 0.74. Additionally, we found the median mortality rate in countries in the top 20 thermal spring revenue group was 2.79 (IQR 1.14 – 3.79) compared to a median mortality rate of 0.09 (IQR 0.02-0.38) in the countries outside the top 20 [figure 2] (p < 0.001).

**Conclusion:**

Our findings support the hypothesis that the high rate of legionella mortality in Japan may be related to the high density of thermal hot springs and spas. These findings further support targeting legionella prevention in these settings, particularly as the wellness industry in this domain continues to grow.

**Disclosures:**

All Authors: No reported disclosures

